# Activated carbon from pistachio shells: A promising approach to improve health metrics in mice with zearalenone exposure

**DOI:** 10.1016/j.toxrep.2025.102197

**Published:** 2025-12-26

**Authors:** Faezeh Oskoueian, Kimia Asadi, Zeinab Javanshir, Saeed Heidarisani, Samira Bozorgi Kasgari, Helia Ghafaripour, Ehsan Karimi, Ehsan Oskoueian

**Affiliations:** a Faculty of Veterinary Medicine, Ferdowsi University of Mashhad, Mashhad, Iran; b Department of Biology, Ma. C., Islamic Azad University, Mashhad, Iran; c Department of Biology, Ferdowsi University of Mashhad, Mashhad, Iran; d Department of Research and Development, Arka Industrial Cluster, Mashhad, Iran; e Department of Animal Science, Khorasan Razavi Agricultural and Natural Resources Research and Education Center (AREEO), Mashhad, Iran

**Keywords:** Biodetoxification, Toxin binder, Mycotoxin, Adsorbents, Fusarium

## Abstract

Zearalenone (ZEN), an estrogenic mycotoxin produced by Fusarium species, poses persistent challenges to food and feed safety due to its potent toxicological effects and widespread occurrence in cereal-based products. This study investigated the protective efficacy of activated carbon (AC) derived from pistachio shells, an abundant agricultural by-product against ZEN-induced toxicity in mice. Pistachio shell carbon was prepared through chemical activation and comprehensively characterized using SEM, FTIR spectroscopy, and BET surface area analysis. Activation increased surface area from 4.1 to 275.9 m²/g and enhanced pore volume from 0.009 to 0.26 cm³/g, confirming substantial improvements in adsorptive architecture. Forty BALB/c mice were assigned to four dietary treatments: control, ZEN-contaminated diet, and ZEN diets supplemented with non-activated or activated carbon (0.5 g/kg). ZEN exposure markedly reduced weight gain, feed intake, antioxidant enzyme activity (GPx, SOD, CAT), and jejunal villus morphology, while elevating serum ALT, AST, and ALP activities. Supplementation with activated carbon significantly ameliorated these adverse effects, restoring growth performance, improving antioxidant status, normalizing liver enzymes, and enhancing intestinal integrity. Histopathological observations corroborated reduced mucosal damage in AC-treated mice. These findings demonstrate that pistachio shell–based activated carbon is a potent, sustainable, and low-cost mycotoxin adsorbent capable of mitigating ZEN toxicity. The study highlights a promising strategy for simultaneous agricultural waste valorization and enhancement of food and feed safety systems.

## Highlights

• Activated carbon derived from pistachio shells serves as a beneficial feed additive.
• Dietary inclusion of pistachio shells improved serum redox potential, and jejunum characteristics in mice on a ZEA-contaminated diet.
• Pistachio shell-based adsorbent mitigated intestinal damage and promoted sustainable detoxification.

## 1 Introduction

Mycotoxins are low-molecular-weight secondary metabolites produced by filamentous fungi, primarily species belonging to the genera *Aspergillus*, *Penicillium*, and *Fusarium*. These toxins remain a major concern for food and feed safety due to their diverse and often insufficiently characterized absorption, distribution, metabolism, excretion, and toxicity properties, which contribute to their complex toxicological behavior [1]. Exposure to mycotoxin-contaminated products has been associated with carcinogenesis, immunosuppression, endocrine disruption, reproductive abnormalities, neurotoxicity, and impaired development in both humans and animals [2], [3]

Among the major regulated mycotoxins, aflatoxins, zearalenone (ZEN), deoxynivalenol (DON), fumonisins, and ochratoxin A (OTA) remain the most common contaminants in agricultural commodities worldwide [4]. ZEN, a potent non-steroidal estrogenic mycotoxin produced predominantly by *Fusarium graminearum*, *F. culmorum*, *F. equiseti*, and *F. semitectum*, is particularly problematic in cereal-based feed and food chains [5]. Its documented effects include reproductive toxicity, neurotoxicity, and carcinogenic potential, leading international organizations such as the International Agency for Research on Cancer to classify ZEN as a possible carcinogenic risk to humans [6]. Consequently, global regulatory authorities have established maximum permissible concentrations of ZEN in food and feed matrices, generally ranging from 1 to 200 µg/kg depending on species, commodity, and regional standards [7].

Efforts to mitigate mycotoxin exposure rely on physical, chemical, and biological detoxification strategies [1]. Among these, physical adsorption is considered advantageous due to its cost-effectiveness, operational simplicity, and minimal adverse effects on the nutritional quality of feed. Recent advances highlight the efficiency of carbon-based materials, modified biochars, and porous adsorbents in capturing mycotoxins from complex matrices [8]. Activated carbon (AC), in particular, exhibits exceptional sorption capability owing to its high surface area and tunable porosity. Chemical modification of AC surfaces can further enhance adsorption selectivity for mycotoxins such as ZEN and OTA, making AC one of the most widely studied and effective sorbents for feed safety applications [9].

Iran, as one of the world’s leading producers of pistachios, generates substantial quantities of pistachio shell waste, accounting for nearly 35–45 % of the harvested crop weight. Due to their high lignocellulosic and carbonaceous content, pistachio shells present an ideal renewable feedstock for the production of high-quality activated carbon [4]. Utilizing this abundant agricultural by-product not only supports circular-economy initiatives but also offers a sustainable pathway for producing adsorbents tailored for mycotoxin remediation. Therefore, the present study aims to compare the efficacy of activated and non-activated carbon derived from pistachio shells in improving feed safety and physiological health parameters in mice exposed to ZEN-contaminated diets. This work highlights the synergistic potential of agricultural waste valorization and advanced mycotoxin management, positioning pistachio shell-based AC as a promising material for food and feed detoxification systems.

## 2 Material and method

### 2.1 Preparation of activated carbon (AC)

Pistachio shells were collected from a commercial pistachio processing facility in Mashhad, Iran, washed, dried at 105 °C for 24 h, and ground to an average particle size of 0.3 mm. Activation was carried out using a standardized chemical activation method following Mirzaie et al. [10] with additional methodological transparency. Briefly, dried biomass was carbonized at 450 °C for 2 h under nitrogen flow (150 mL/min). The carbonized material was then impregnated with activating agents at a 1:10 (w/v) solid-to-solution ratio using H₂SO₄ (7 M), HNO₃ (5 M), NH₃ (6 M), or NaOH (6 M). The suspensions were maintained at 70 °C for 2 h with continuous stirring and subsequently shaken at 30 °C for 24 h. After treatment, samples were repeatedly washed with deionized water until a neutral pH was achieved and then oven-dried at 120 °C for 4 h. Both activated and non-activated carbons were prepared in three independent replicates and pooled into a homogeneous stock prior to diet formulation to reduce batch variation.

### 2.2 Physicochemical characterization of AC

A comprehensive characterization was performed to assess structural and functional properties relevant to ZEN adsorption capacity. Surface morphology was observed using scanning electron microscopy (SEM) under an argon atmosphere [11]. Surface area, total pore volume, and average pore diameter were determined using N₂ adsorption–desorption isotherms and calculated via the BET method [12]. Functional groups were analyzed by FTIR spectroscopy between 3500 and 500 cm⁻¹ (Ogungbenro et al., 2020). All analyses were performed in triplicate and reported as mean ± SD.

### 2.3 Production and extraction of zearalenone (ZEN)

A substrate mixture (45 % corn flour, 20 % soybean meal, 15 % wheat flour, 20 % wheat bran) was prepared, and 500 g of the mixture was distributed into ten 2000-mL Erlenmeyer flasks to serve as biological replicates. After hydration with 250 mL sterile distilled water, flasks were autoclaved at 120 °C for 15 min. A confirmed ZEN-producing Fusarium graminearum isolate (Tarbiat Modares University Culture Collection; ID: TMU-Fg-ZEN001) was grown for 7 days on potato dextrose agar (PDA) and used for solid-state fermentation following Safaei et al. [13]. Flasks were incubated at 25 °C for 20 days. Fermented material (400 g) was extracted with 2000 mL of 80 % methanol (v/v), shaken for 3 h, filtered, and concentrated under reduced pressure. The dried extract was dissolved in DMSO. ZEN concentration was quantified by LC-MS, following the method described by Hsu et al. [14]. Extraction was performed in triplicate, and ZEN concentration was standardized across batches.

### 2.4 Animal experiment

All procedures were performed in accordance with the Institutional Animal Care and Use Committee (IACUC) guidelines and were approved under the protocol of Islamic Azad University, Mashhad Branch. Forty male BALB/c mice (20–25 g) were randomly assigned using a computer-generated randomization sequence into four treatment groups (n = 10 per group). Mice were individually housed under controlled environmental conditions (23 ± 1 °C; humidity 58 ± 10 %; 12 h light/dark cycle) with ad libitum access to feed and water. Dietary treatments were: T1: Basal diet; T2: Basal diet + ZEN (10 mg/kg diet); T3: ZEN (10 mg/kg) + non-activated pistachio-shell carbon (0.5 g/kg); T4: ZEN (10 mg/kg) + activated pistachio-shell carbon (0.5 g/kg). Feed intake and body weight were recorded weekly. On day 28, mice were euthanized using intraperitoneal pentobarbital-HCl (50 mg/kg). Blood samples were collected via cardiac puncture; jejunum and liver were harvested, snap-frozen, or fixed depending on analysis requirements.

### 2.5 Blood biochemistry and antioxidant assays

Serum AST, ALT, ALP, CAT, GPx, SOD, IgA, IgM, and IgG were analyzed using an automated chemistry analyzer following procedures described by Nassef [15]. All measurements were conducted in triplicate.

### 2.6 Histopathology and intestinal morphometrics

Liver and jejunum tissues were fixed in 10 % neutral-buffered formalin (pH 7.0), processed, paraffin-embedded, and sectioned at 5 µm. Hematoxylin-eosin staining was performed following previous research [16]. Slides were examined under a light microscope (20 × magnification). Villus height, villus width, and crypt depth were measured from 10 well-oriented villi per sample.

### 2.7 Gene expression analysis

Liver tissues were homogenized in liquid nitrogen, and total RNA was extracted using the RNeasy Mini Kit (Qiagen, Germany). cDNA synthesis employed the QuantiTect RT Kit (Qiagen). qPCR was performed using an initial denaturation at 95 °C for 5 min, followed by 35 cycles of 95 °C (20 s), 60 °C (30 s), and 72 °C (30 s). Expression of SOD, iNOS, and IFN-γ was normalized to β-actin using the 2⁻ΔΔCt method. Primers are listed in Table 1.

**Table 1:** The primer sets characteristics used in this study.

Gene	Forward (5` →3`)	Reverse (5` →3`)	References
SOD	gagacctgggcaatgtgact	gtttactgcgcaatcccaat	[17]
iNOS	caccttggagttcacccagt	accactcgtacttgggatgc	[18]
IFN-γ	tgagctcattgaatgcttgg	ccaacacccagtgacgacc	[19]
β-actin	cctgaaccctaaggccaacc	cagctgtggtggtgaagctg	[20]

### 2.8 Statistical analysis

Data were analyzed using SAS software (Version 9.4, SAS Institute Inc., Cary, NC, USA). A completely randomized design (CRD) was applied. The GLM procedure was used to determine the effects of dietary treatments.

Model: Yᵢⱼ = µ + Tᵢ + εᵢⱼ Where:

µ = overall mean,

Tᵢ = treatment effect,

εᵢⱼ = residual error.

Assumptions of normality and homogeneity of variances were tested using Shapiro-Wilk and Levene’s tests, respectively. When a significant treatment effect was observed (p < 0.05), Duncan’s multiple range test was chosen because of its suitability for nutritional studies where the detection of inter-group differences with moderate sample size is required. Results are presented as mean ± SEM, with F-statistics and exact p-values reported for all analyzed parameters.

## 3 Results and discussion

### 3.1 Scanning electron microscopy

Fig. 1 presents the SEM micrographs showing the microstructure of activated carbon. The images clearly depict pore formation with cavities of varying sizes and shapes across the activated carbon surface. While mechanistic explanations regarding graphitic layer degradation cannot be conclusively inferred from SEM alone, the observed porous morphology is consistent with structural changes expected during alkaline activation processes, such as NaOH-induced surface etching and partial oxidation. These visually identifiable micro- and mesopores align with known structural outcomes of chemical activation, although direct confirmation would require complementary molecular or adsorption analyses (e.g., BET, elemental mapping, or Raman spectrometry).

**Fig. 1:**
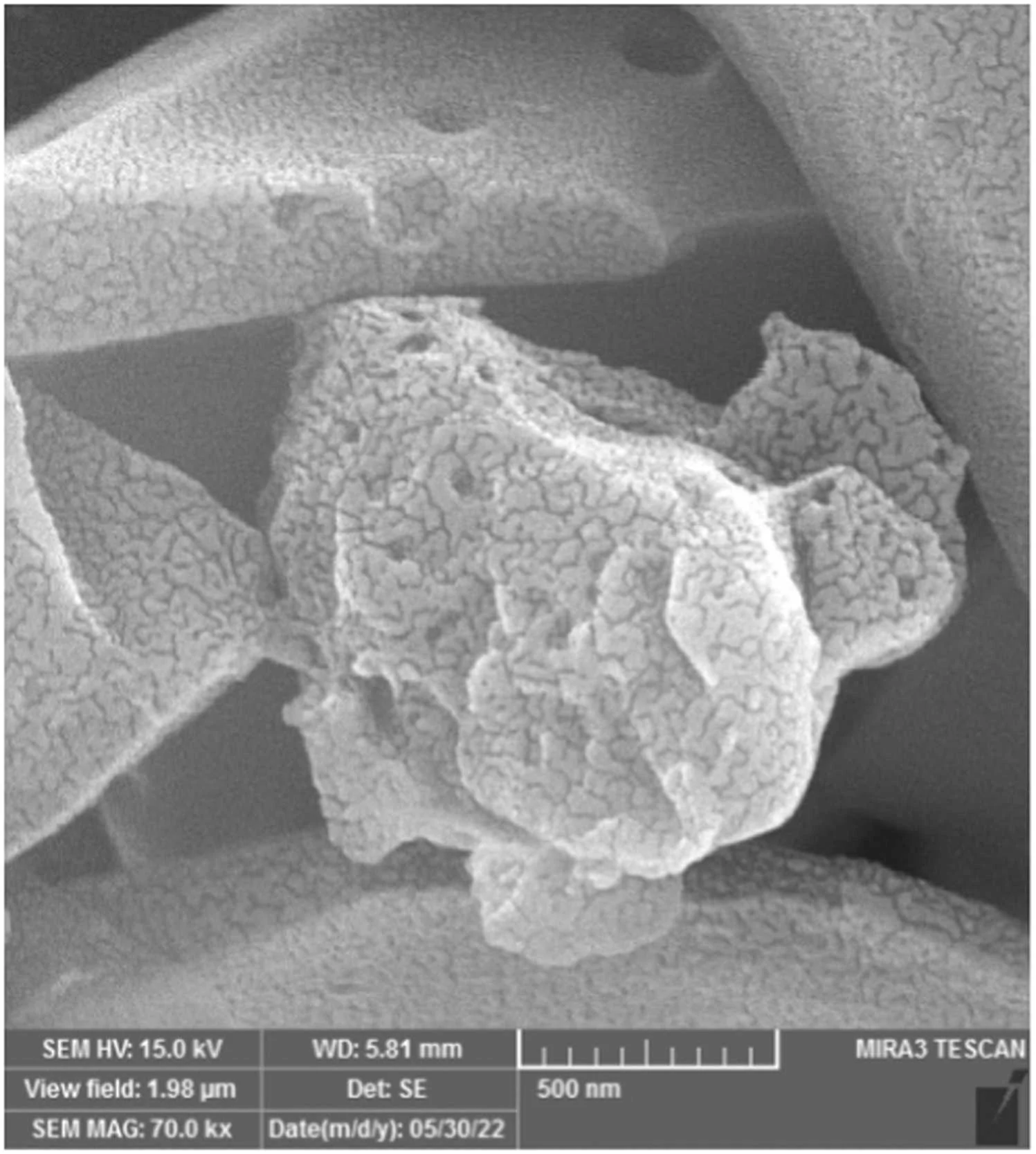
Scanning electron micrograph (SEM) of the activated carbon.

### 3.2 Physicochemical characterization of activated carbon

Structural parameters of the activated carbon are summarized in Table 2. The specific surface area and pore volume increased significantly (P < 0.05) after activation, rising from 4.1 to 275.9 m²/g for surface area and from 0.009 to 0.26 cm³ /g for pore volume. Although the mechanisms behind pore formation cannot be fully characterized without more detailed adsorption modeling, these values are in line with typical increases observed following alkaline activation. Previous studies have reported similar trends, showing that activation enhances micro- and mesopore abundance, improving capacity for physical adsorption of mycotoxins. The mean pore diameter decreased following activation, consistent with the development of smaller pores. This structural shift suggests an enhanced potential for interaction with low-molecular-weight toxins such as ZEN, although adsorption specificity cannot be confirmed in the absence of isotherm or kinetic data.

**Table 2:** Physical characteristics of pistachio shell-derived non-activated and activated carbon materials.

Parameters	Inactive carbon	Activated carbon	SEM
Specific surface area (m^2^/g)	4.1^b^	275.9^a^	9.32
Total pore volume (cm^3^/ g)	0.009^b^	0.26^a^	0.01
Mean pore diameter (nm)	48.7^a^	10.2^b^	4.72

### 3.3 FTIR analysis

The chemical structure of surface functional groups in activated carbon (AC) can significantly influence adsorption behavior, although adsorption mechanisms cannot be fully inferred from FTIR alone. As such, the FTIR spectra for non-activated carbon and activated carbon are shown in Fig. 2 for comparative purposes. In both samples there are broad peaks around ∼3428 cm⁻¹ (inactive carbon) and ∼3424 cm⁻¹ (activated carbon), as well as at ∼1110 cm⁻¹, which may be attributed to C–H stretching or bending modes. Very weak bands near ∼2100 cm⁻¹ and ∼2300 cm⁻¹ are present in both materials and may correspond to carbon–oxygen double bonds or ketone/quinone-type groups [21].

**Fig. 2:**
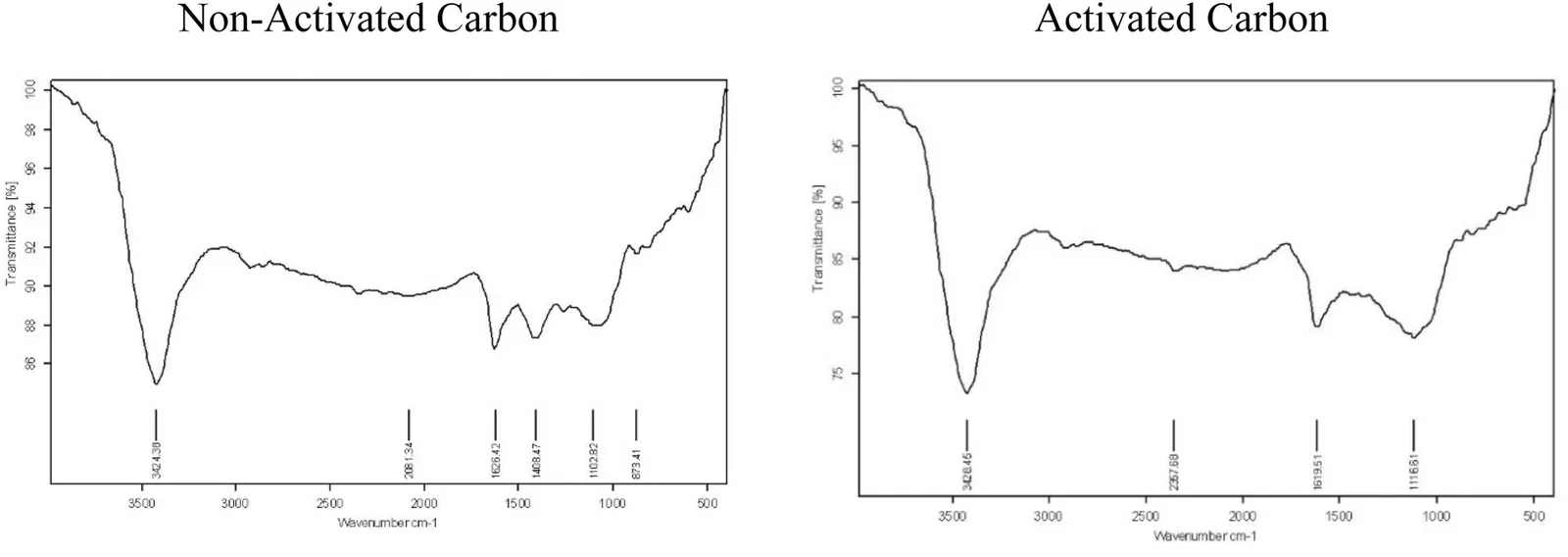
FTIR spectra of non-activated and activated pistachio shell-based carbons; scale marks at 500 cm⁻¹ intervals; intensity (a.u.) vs wavenumber (cm⁻¹).

Importantly, the activated carbon shows a distinct and more intense band at ∼1520 cm⁻¹, which is commonly assigned to C=O stretching of carboxyl or lactone groups indicative of oxidation via nitric acid. The increased intensity of this band suggests a higher concentration of acidic oxygenated functional groups on the surface after activation. These groups may contribute to enhanced adsorption of certain molecules through hydrogen-bonding or electrostatic interactions, although no adsorption isotherm or mechanistic binding study is provided here, so any interpretation remains correlative, not causal.

From the literature, it is known that the presence of oxygen- and nitrogen-containing surface groups on activated carbons can enhance the adsorption of polar molecules by increasing surface polarity and providing specific interaction sites [22]. Another study showed that carboxyl and carbonyl groups on carbon surfaces improved the adsorption of volatile compounds via non-covalent interactions [23]. Although that work focused on gas adsorption, the principle of enhanced interactive potential through functional groups is relevant to mycotoxin adsorption as well. Therefore, in the present case, the appearance of enhanced C=O bands in the activated carbon suggests that surface chemistry modification occurred and may very well contribute to adsorption potential. However, in the absence of direct adsorption kinetics or binding affinity data (e.g., ZEN binding isotherms), we refrain from stating a mechanistic chain of “activation → more acidic groups → greater toxin binding → improved in vivo outcome”. Instead, we state that the increased oxygenated functional groups may enhance adsorption by providing additional interactive sites, but further work is required to verify this.

### 3.4 Weight gain and feed intake

Essential parameters measured in experimental animals to assess their growth and nutritional status are feed intake and weight gain. Weight gain refers to the increase in body weight over a set timeframe, while feed intake is the quantity of food consumed in that timeframe [24]. These measurements play an important role in investigations of how different diets, interventions or treatments impact animal physiology and metabolism, because changes in feed intake and weight gain may suggest variations in nutrient utilization efficiency, appetite regulation, or energy status [24], [25].

The results, shown in Table 3, indicate that dietary contamination with Zearalenone (ZEN) significantly (p < 0.05) reduced average daily weight gain and average daily food intake compared with the control diet. Supplementation with non-activated carbon significantly (p < 0.05) improved both parameters relative to the ZEN-only group, while activated carbon produced the greatest improvement in both daily weight gain and food intake.

**Table 3:** Effects of dietary pistachio shell-derived non-activated and activated carbon on average daily weight gain and average daily feed intake in mice fed a zearalenone-contaminated diet.

Parameters	T1	T2	T3	T4	SEM
Average daily weight gain (mg/day)	142.5^a^	95.6^d^	109.3^c^	125.7^b^	3.62
Average daily food intake (g/day)	4.3^a^	3.2^d^	3.5^c^	3.9^b^	0.14

T1: mice fed a standard (control) diet.

T2: mice fed a zearalenone-contaminated diet (10 mg/kg).

T3: mice fed a zearalenone-contaminated diet (10 mg/kg) supplemented with pistachio shell–derived non-activated carbon (0.5 g/kg).

T4: mice fed a zearalenone-contaminated diet (10 mg/kg) supplemented with pistachio shell–derived activated carbon (0.5 g/kg).

Values within the same row bearing different superscript letters differ significantly (p < 0.05).

Sample size: n = 10; experimental period: Days 0–28.

These findings align with recent reports showing that ZEN-contaminated diets reduce animal growth performance by affecting feed consumption and nutrient assimilation [26]. Diet composition affects long-term ZEN exposure. For instance, Thapa et al. [26] documented that ZEN exposure in mice decreased feed intake and weight gain, underscoring the anorectic and growth-inhibitory effects of this mycotoxin. Accordingly, our finding that activated carbon improves these parameters suggests an ameliorative effect on ZEN’s negative impact on growth.

However, a mechanistic interpretation must be cautious: while it is tempting to infer that activated carbon binds ZEN in the gut and thereby restores nutrient intake and growth, we did not directly measure ZEN concentrations in the gut lumen or blood nor perform adsorption isotherm studies. Therefore, the improved growth cannot be definitively attributed to toxin binding. The observed improvements may equally reflect reduced toxin bioavailability, improved feed palatability, improved gut morphology (as shown later), or a combination thereof.

### 3.5 Blood biochemical parameters

Blood biochemical indices serve as essential indicators of physiological status and are widely applied in both experimental and clinical diagnostics [27]. Liver-associated enzymes alanine aminotransferase (ALT), alkaline phosphatase (ALP), and aspartate aminotransferase (AST) are predominantly localized in hepatocytes; therefore, their elevated serum concentrations typically reflect hepatic injury or impaired liver function (Kalas et al., 2021). Similarly, antioxidant enzymes such as superoxide dismutase (SOD), catalase (CAT), and glutathione peroxidase (GPx) constitute key components of the endogenous antioxidant defense system, and reductions in their activity are indicative of oxidative stress and compromised redox balance [28]. Monitoring these parameters provides critical insight into toxicological responses, including hepatic damage, oxidative imbalance, and related systemic alterations.

The blood biochemical markers evaluated in this study, liver enzymes (Table 4), immunoglobulins (Table 5), and antioxidant enzymes (Table 6) demonstrated clear responses to dietary zearalenone exposure. Feeding mice with a zearalenone-contaminated diet resulted in a significant (p < 0.05) elevation of serum AST, ALT, and ALP activities, confirming the hepatotoxic nature of zearalenone reported previously in rodent and livestock models. Concurrently, activities of the antioxidant enzymes GPx, SOD, and CAT were significantly reduced (p < 0.05), consistent with earlier studies showing that zearalenone induces oxidative stress by generating reactive oxygen species and impairing enzymatic defenses.

**Table 4:** Effects of dietary pistachio shell–derived non-activated and activated carbon on serum liver enzyme activities in mice fed a zearalenone-contaminated diet.

**Treatments**	**AST** **(U/L)**	**ALT** **(U/L)**	**ALP** **(U/L)**
**T1**	135^d^	101^d^	156^d^
**T2**	165^a^	147^a^	191^a^
**T3**	151^b^	134^b^	182^b^
**T4**	144^c^	118^c^	163^c^
**SEM**	3.39	4.11	3.43

T1: mice fed a standard (control) diet.

T2: mice fed a zearalenone-contaminated diet (10 mg/kg).

T3: mice fed a zearalenone-contaminated diet (10 mg/kg) supplemented with pistachio shell–derived non-activated carbon (0.5 g/kg).

T4: mice fed a zearalenone-contaminated diet (10 mg/kg) supplemented with pistachio shell–derived activated carbon (0.5 g/kg).

Values within the same column bearing different superscript letters differ significantly (p < 0.05)

**Table 5:** Effects of dietary pistachio shell-derived non-activated and activated carbon on serum immunoglobulin concentrations in mice fed a zearalenone-contaminated diet.

**Treatment**	**IgA (mg/dl)**	**IgG (mg/dl)**	**IgM (mg/dl)**
**T1**	7.3^a^	7.9^a^	7.3^a^
**T2**	6.1^c^	6.1^c^	5.2^d^
**T3**	7.1^ab^	6.5^bc^	5.9^c^
**T4**	7.4^a^	6.9^b^	6.6^b^
SEM	0.14	0.08	0.10

T1: mice fed a standard (control) diet.

T2: mice fed a zearalenone-contaminated diet (10 mg/kg).

T3: mice fed a zearalenone-contaminated diet (10 mg/kg) supplemented with pistachio shell–derived non-activated carbon (0.5 g/kg).

T4: mice fed a zearalenone-contaminated diet (10 mg/kg) supplemented with pistachio shell–derived activated carbon (0.5 g/kg).

Values within the same column bearing different superscript letters differ significantly (p < 0.05)

**Table 6:** Effects of dietary pistachio shell-derived non-activated and activated carbon on serum antioxidant enzyme activities in mice fed a zearalenone-contaminated diet.

**Treatment**	**GPX** **(U/mL)**	**SOD** **(U/mL)**	**CAT** **(U/mL)**
**T1**	588^a^	56^a^	37^a^
**T2**	402^d^	33^d^	22^d^
**T3**	465^c^	38^c^	28^c^
**T4**	504^b^	47^b^	32^b^
**SEM**	4.73	2.11	2.06

T1: mice fed a standard (control) diet.

T2: mice fed a zearalenone-contaminated diet (10 mg/kg).

T3: mice fed a zearalenone-contaminated diet (10 mg/kg) supplemented with pistachio shell–derived non-activated carbon (0.5 g/kg).

T4: mice fed a zearalenone-contaminated diet (10 mg/kg) supplemented with pistachio shell–derived activated carbon (0.5 g/kg).

Values within the same column bearing different superscript letters differ significantly (p < 0.05)

Dietary inclusion of inactive carbon partially mitigated these adverse effects by lowering liver enzyme activities and enhancing antioxidant enzyme levels. However, activated carbon exhibited markedly superior protective effects, leading to more pronounced normalization of hepatic markers and restoration of antioxidant enzyme activities. This improved efficacy aligns with recent reports demonstrating the higher adsorption capacity, surface area, and porosity of activated carbon, which enhance its ability to bind and neutralize mycotoxins in the gastrointestinal tract.

No significant differences were observed in serum immunoglobulin concentrations among treatments, suggesting that the dietary ZEA levels and carbon-based adsorbents primarily affected hepatic and oxidative pathways rather than systemic humoral immunity. This observation is consistent with previous findings indicating that immunoglobulin responses to ZEA are generally less sensitive than oxidative and hepatic biomarkers under comparable exposure conditions.

### 3.6 Histopathological examination and morphometric analysis

Histopathological evaluation revealed no observable hepatic lesions in mice receiving the mycotoxin-contaminated diets (T2, T3, and T4) compared with the control group, as illustrated in Fig. 3. Although the absence of visible hepatic alterations may appear unexpected, previous studies have shown that the severity of zearalenone (ZEN)-induced liver damage depends strongly on dose, exposure duration, and animal sensitivity. Nonetheless, extensive literature confirms that ZEN is capable of inducing hepatic degeneration, inflammatory infiltration, and architectural disruption through mechanisms driven largely by oxidative stress and inflammatory signaling [29], [30]. In line with the biochemical findings of elevated liver enzymes reported earlier in this study, these results suggest that functional hepatic impairment may occur even when overt morphological lesions are not yet detectable.

**Fig. 3:**
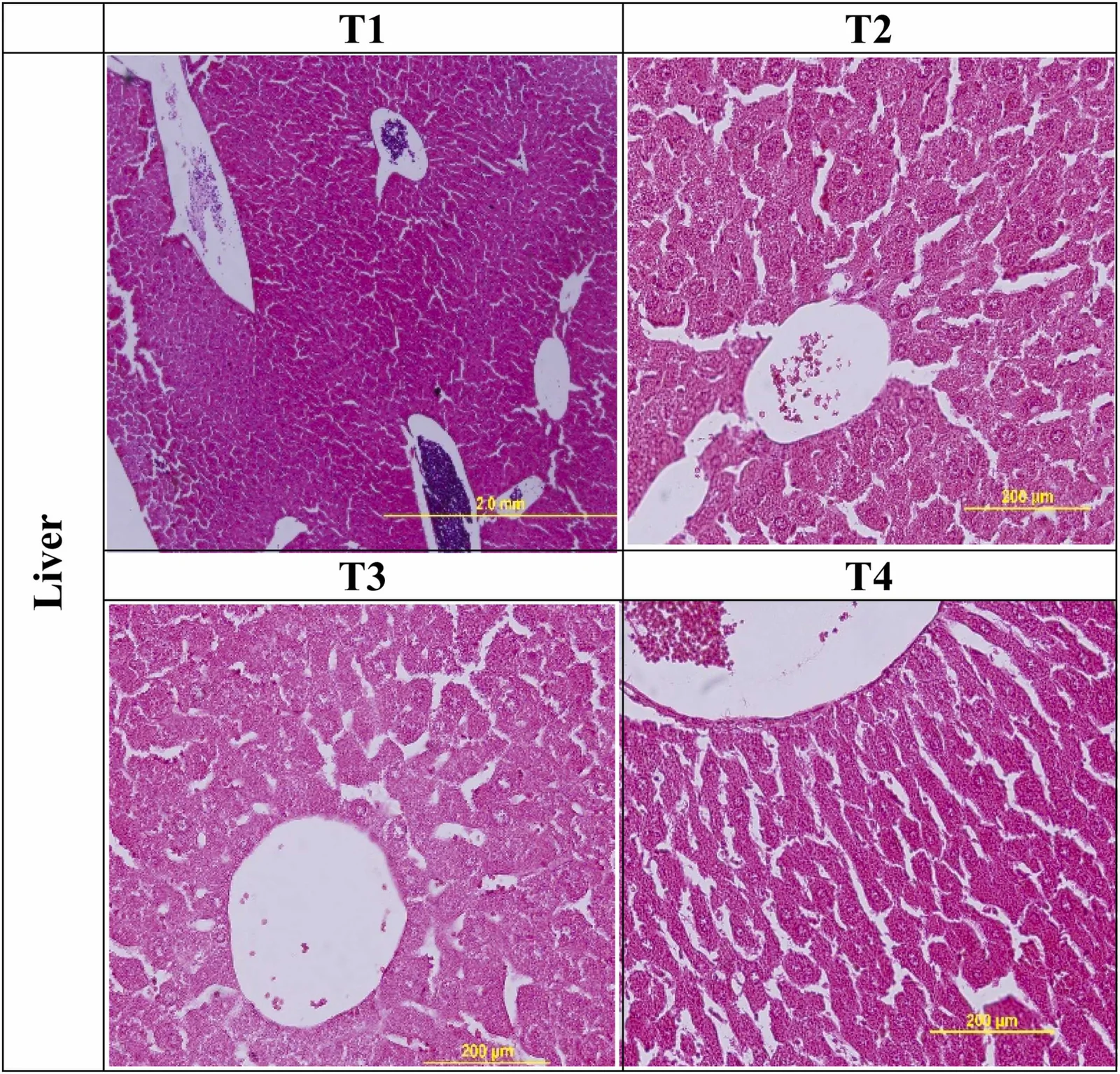
Histopathological evaluation of jejunum in mice fed a zearalenone-contaminated diet, illustrating the impact of dietary supplementation with pistachio shell–based non-activated and activated carbon.

The supplementation of activated carbon appeared to confer a protective effect against potential hepatic damage. Its high surface area and porous structure likely enhanced its adsorption efficiency, reducing ZEN bioavailability within the gastrointestinal tract and thereby limiting systemic distribution. These findings support earlier reports demonstrating the superior binding capacity of activated carbon among commonly used mycotoxin adsorbents.

Morphometric assessment of the jejunum provides critical insight into the structural integrity of the intestinal mucosa [31]. Changes in villus height, villus width, and crypt depth serve as sensitive indicators of intestinal injury caused by mycotoxin exposure. As presented in Table 7, dietary ZEN (T2) significantly (p < 0.05) reduced villus height and width while altering crypt depth, indicating compromised mucosal architecture and diminished absorptive capacity. Such structural deterioration is consistent with ZEN’s known effects on epithelial turnover, nutrient absorption, and gut barrier function.

**Table 7:** Effects of dietary pistachio shell–derived non-activated and activated carbon on jejunal morphometric characteristics in mice fed a zearalenone-contaminated diet.

	**Villus Height (µm)**	**Villus Width (µm)**	**Crypt Depth (µm)**
**T1**	623^a^	123^a^	204^d^
**T2**	426^d^	85^d^	278^a^
**T3**	451^c^	93^c^	253^b^
**T4**	537^b^	106^b^	226^c^
SEM	6.38	3.18	6.03

T1: Mice fed a normal diet

T2: Mice fed a zearalenone-contaminated diet (10 mg/kg diet)

T3: Mice fed a zearalenone-contaminated diet (10 mg/kg diet) supplemented with pistachio shell–based non-activated carbon (0.5 g/kg diet)

T4: Mice fed a zearalenone-contaminated diet (10 mg/kg diet) supplemented with pistachio shell–based activated carbon (0.5 g/kg diet)

Note: Different letters within the same column indicate statistically significant differences (p < 0.05).

Dietary inclusion of carbon, particularly activated carbon, effectively counteracted these adverse morphological alterations. Activated carbon supplementation significantly improved villus height, villus width, and crypt depth (p < 0.05), reflecting restoration of mucosal integrity and enhanced nutrient absorptive potential. These improvements align with the observed increases in average daily weight gain and feed intake (Table 2), suggesting that intestinal recovery translates into measurable physiological benefits.

Overall, the morphometric and histopathological findings collectively demonstrate that activated carbon provides superior protection against ZEN-induced hepatic and gastrointestinal alterations by reducing toxin bioavailability and preserving tissue structure.

### 3.7 Gene’s profiling pattern

Gene expression profiling provides deeper insight into the molecular mechanisms through which zearalenone (ZEN) and carbon-based adsorbents influence oxidative stress, inflammation, and immune modulation. The superoxide dismutase (SOD) genes play a central role in the antioxidant defense system by catalyzing the dismutation of superoxide radicals [32], whereas inducible nitric oxide synthase (iNOS) is a key pro-inflammatory marker strongly associated with oxidative and inflammatory injury [33]. Interferon-gamma (IFN-γ), a major cytokine produced by activated T cells and natural killer cells, is critical for macrophage activation and the coordination of both innate and adaptive immune responses [34].

In the present study, dietary ZEN exposure significantly downregulated (p < 0.05) the expression of SOD and IFN-γ genes while upregulating (p < 0.05) iNOS expression (Table 8). This expression pattern is consistent with the biochemical findings, where ZEN impaired antioxidant enzyme activity and promoted oxidative and inflammatory stress. The suppression of SOD and IFN-γ indicates weakened antioxidant capacity and reduced immune activation, whereas increased iNOS expression reflects heightened inflammatory signaling patterns widely documented in ZEN-induced toxicity models.

**Table 8:** Effects of dietary pistachio shell-derived non-activated and activated carbon on the expression of antioxidant-, inflammatory-, and immunomodulatory-related genes in mouse jejunal tissue.

**Gene expression (Fold changes)**					S.E.M
**Genes**	**T1**	**T2**	**T3**	**T4**	
**SOD**	1	-3.6	-3.1	-2.4	0.42
**iNOS**	1	+4.2	+3.5	+2.6	0.31
**IFN-γ**	1	-2.7	-2.1	-1.4	0.25

T1: Mice fed a normal diet

T2: Mice fed a zearalenone-contaminated diet (10 mg/kg diet)

T3: Mice fed a zearalenone-contaminated diet (10 mg/kg diet) supplemented with pistachio shell–based non-activated carbon (0.5 g/kg diet)

T4: Mice fed a zearalenone-contaminated diet (10 mg/kg diet) supplemented with pistachio shell–based activated carbon (0.5 g/kg diet)

Different letters within the same row indicate statistically significant differences (p < 0.05).

“−” denotes down-regulation relative to the control group (T1).

“+ ” denotes up-regulation relative to the control group (T1).

Supplementation with carbon, particularly activated carbon, demonstrated a strong protective effect at the molecular level. Activated carbon significantly upregulated SOD and IFN-γ expression (p < 0.05) and concurrently suppressed iNOS expression (p < 0.05). These results support the superior binding efficacy of activated carbon, which likely reduces ZEN’s intestinal absorption and systemic exposure. By limiting toxin bioavailability, activated carbon mitigates oxidative stress, restores antioxidant gene expression, and enhances immune regulation. This aligns with earlier findings suggesting that activated carbon can attenuate mycotoxin-induced inflammation and modulate cytokine expression toward a more balanced immune response [35].

Overall, the gene expression data substantiate the biochemical and morphometric improvements observed in other sections of this study, highlighting activated carbon as an effective dietary strategy for alleviating ZEN-induced oxidative and inflammatory damage.

## 4 Conclusion

This study demonstrates that activated carbon derived from pistachio shells is a highly effective and sustainable intervention for mitigating zearalenone-induced toxicity in mammals. Chemical activation profoundly enhanced the physicochemical properties of pistachio shell carbon, yielding a material with markedly increased surface area, pore volume, and functional surface groups capable of adsorbing low-molecular-weight mycotoxins such as ZEN. In vivo findings revealed that dietary AC supplementation significantly alleviated the detrimental effects of ZEN on growth performance, liver function, oxidative balance, and intestinal morphology. Activated carbon outperformed non-activated carbon across all biological indices, underscoring the importance of tailored physicochemical engineering for optimal detoxification efficacy.

The ability of pistachio shell–based AC to restore antioxidant enzyme activity, normalize hepatic biomarkers, and improve villus architecture positions it as a promising candidate for next-generation mycotoxin mitigation strategies. Moreover, valorizing pistachio shells a major agro-industrial waste in Iran and other pistachio-producing regions, provides a circular-economy solution with tangible environmental and economic benefits. Future studies should further explore adsorption kinetics, in vivo binding stability, and application in livestock production systems. Collectively, these findings establish activated carbon derived from pistachio shells as a potent, eco-friendly, and scalable adsorbent with significant implications for food and feed safety.

## CRediT authorship contribution statement

**Helia Ghafaripour:** Methodology, Investigation. **Samira Bozorgi Kasgari:** Methodology, Investigation. **Saeed Heidarisani:** Methodology, Investigation. **Zeinab Javanshir:** Methodology, Investigation. **Ehsan Oskoueian:** Writing – review & editing, Visualization, Validation, Software. **Ehsan Karimi:** Writing – review & editing, Supervision, Project administration. **Faezeh Oskoueian:** Investigation. **Kimia Asadi:** Methodology, Investigation.

## Ethical approval

This research does not contain any studies with human participants performed by any of the authors. All animal experiments were conducted according to the ethical principles approved by the Islamic Azad University of Mashhad.

## Consent to participate

NA/Not applicable.

## Consent to publication

Not applicable.

## Funding

There has been no financial support for this work.

## Declaration of Competing Interest

The authors declare that they have no known competing financial interests or personal relationships that could have appeared to influence the work reported in this paper.

## Data Availability

Data will be made available on request.
